# Molecular approaches drive the raceCAR team against cancer

**DOI:** 10.1016/j.omton.2025.201113

**Published:** 2025-12-26

**Authors:** Dean A. Lee

**Affiliations:** 1 Center for Childhood Cancer Research, The Abigail Wexner Research Institute, Nationwide Children’s Hospital, Columbus, OH, USA

## Main text

We are pleased to share this special collection of articles on T cell and natural killer (NK) cell immunotherapy from just the past 2 years of MTO. Though covering such a short time frame, these 42 research articles and 4 reviews nicely illustrate the current state of the field. It might be overly simplistic (or even self-obvious) that, in order to be effective, cellular therapy must be able to traffic to tumor sites (4 articles addressing homing), persist in a toxic environment (8 articles on TME including TGFb), recognize an antigen sufficiently expressed on the tumor (15 articles on novel targets, combinations, or enhancing target expression) but with minimized recognition of healthy tissue (6 articles on mitigating toxicity), and have optimal killing ability (12 articles on enhancing effector function). Finally, the last few years have taught us that it must be cost-effective (4 articles on manufacturing improvements).

**Figure 1:**
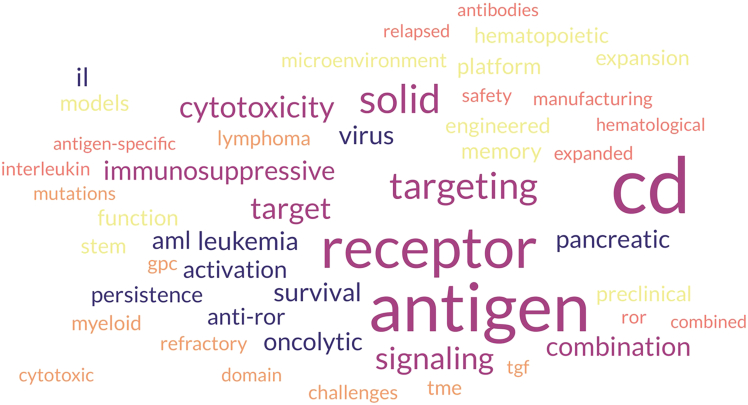
Word cloud generated from the titles of the 42 articles in this collection

To illustrate the themes in a more unbiased way, I created a word-cloud from the collated titles and abstracts of the research articles, removing the obvious criteria for publishing in this journal (molecular, therapies, oncology—after all, this *is* MTO), inclusion in this series (T cells, NK cells, CARs), or otherwise obvious terms (efficacy, model, *vivo*, *vitro*, treatment, novel, improved, enhanced) (Figure 1). Receptors, targeting, and CD antigens might still be in that “self-obvious” category, but common themes of survival, combinations, signaling, immunosuppressive, persistence, and solid (tumors) seem on target.

Other important themes include preventing immune escape by enforcing antigen expression/density, Boolean gating for preventing off-target toxicity, and novel combinations with synergy. Given the history of the journal (previously known as *Molecular Therapy* – *Oncolytics*), it is not surprising that combining cellular therapy with oncolytic viruses is a recurring theme.

Included here are a number of novel engineering and manufacturing methodologies and product platforms designed to improve product quality, efficiency, or performance, novel applications of existing approaches to new settings (Fanconi anemia, using CARs to supplant chemotherapy for conditioning), and novel assays to solve research headaches.

The four reviews in this collection do more than just define the current state of the art; they set the stage for addressing important advances and answering focused problems that need to be solved for more effective cellular therapy. Lastly, the importance of core basic science to identify driving mechanisms of these problems is also evident in this collection, as is a focus on hard-to-treat or rare cancers.

We hope you find this collection engaging and timely. I found that just perusing the titles alone made me curious and thoughtful about new directions in my own research!

